# Muscle Activation Patterns When Passively Stretching Spastic Lower Limb Muscles of Children with Cerebral Palsy

**DOI:** 10.1371/journal.pone.0091759

**Published:** 2014-03-20

**Authors:** Lynn Bar-On, Erwin Aertbeliën, Guy Molenaers, Kaat Desloovere

**Affiliations:** 1 Clinical Motion Analysis Laboratory, University Hospital Leuven, Leuven, Belgium; 2 KU Leuven Department of Rehabilitation Sciences, Leuven, Belgium; 3 KU Leuven Department of Mechanical Engineering, Leuven, Belgium; 4 KU Leuven Department of Development and Regeneration, Leuven, Belgium; 5 Department of Orthopaedics, University Hospital Leuven, Leuven, Belgium; University of Sydney, Australia

## Abstract

The definition of spasticity as a velocity-dependent activation of the tonic stretch reflex during a stretch to a passive muscle is the most widely accepted. However, other mechanisms are also thought to contribute to pathological muscle activity and, in patients post-stroke and spinal cord injury can result in different activation patterns. In the lower-limbs of children with spastic cerebral palsy (CP) these distinct activation patterns have not yet been thoroughly explored. The aim of the study was to apply an instrumented assessment to quantify different muscle activation patterns in four lower-limb muscles of children with CP. Fifty-four children with CP were included (males/females n = 35/19; 10.8±3.8 yrs; bilateral/unilateral involvement n =  32/22; Gross Motor Functional Classification Score I–IV) of whom ten were retested to evaluate intra-rater reliability. With the subject relaxed, single-joint, sagittal-plane movements of the hip, knee, and ankle were performed to stretch the lower-limb muscles at three increasing velocities. Muscle activity and joint motion were synchronously recorded using inertial sensors and electromyography (EMG) from the adductors, medial hamstrings, rectus femoris, and gastrocnemius. Muscles were visually categorised into activation patterns using average, normalized root mean square EMG (RMS-EMG) compared across increasing position zones and velocities. Based on the visual categorisation, quantitative parameters were defined using stretch-reflex thresholds and normalized RMS-EMG. These parameters were compared between muscles with different activation patterns. All patterns were dominated by high velocity-dependent muscle activation, but in more than half, low velocity-dependent activation was also observed. Muscle activation patterns were found to be both muscle- and subject-specific (*p*<0.01). The intra-rater reliability of all quantitative parameters was moderate to good. Comparing RMS-EMG between incremental position zones during low velocity stretches was found to be the most sensitive in categorizing muscles into activation patterns (*p*<0.01). Future studies should investigate whether muscles with different patterns react differently to treatment.

## Introduction

Cerebral Palsy (CP) is the most common neurological disorder in children [1] and is associated with an upper motor neuron lesion occurring in the immature brain. Of the patients with CP, 80–90% are classified as having spasticity. Secondary problems to spasticity include pain, muscle and soft tissue contracture, bony deformities, and as a result of these, increasing limitations in activity and function [2]. Therefore, spasticity management begins at an early age and aims to prevent these secondary impairments [3]. However, there is a large response variability to current spasticity treatment, such as Botulinum Toxin A (BTX) [4], [5]. It is therefore important to correctly assess spasticity, differentiate it from other positive signs of the upper motor neuron syndrome, and try to understand why some children react better than others to tone-reduction treatment. This in turn, will ensure that a child with CP receives therapy tailored to the mechanisms contributing to his or her specific symptoms.

Spasticity is most commonly defined as “a velocity-dependent increase in tonic stretch reflex with exaggerated tendon jerks, resulting from hyper excitability of the stretch reflex, as one component of the upper motor neurone syndrome” [6]. Multiple studies have also shown increased activation when relaxed muscles were stretched at very low velocities [7]–[11] sometimes continuing once the movement had stopped [12]. This suggests the involvement of physiological mechanisms other than activation of the phasic stretch-reflex. One explanation is that changes in the membrane properties of alpha motor neurones increase their sensitivity to weak afferent input, such as that during very low velocity stretch [13]. This in turn triggers persistent inward currents (PIC) that lead to prolonged depolarization states called plateau potentials. Following loss of normal central regulation, PIC and plateaus can result in continuous low-level motor output [13]. These have been found to be related to spasticity in chronic spinal cord injury, [14] and in persons post-stroke [15].Other mechanisms that may potentiate sustained activation could involve group-II muscle spindle afferents that are more sensitive to muscle length than to velocity [12], [16], cutaneous [17] or nociceptive [18] stimulation.

Pandyan et al. observed a variety of muscle activation patterns in the elbow flexors that can be associated with clinical spasticity in subjects post-stroke: (a) an increase in muscle activity during quiet sitting, (b) movement-dependent muscle activity also occurring at stretch velocities <10°/s, and (d) muscle activation patterns consistent with a clasp-knife phenomenon [9]. Similar patterns were reported by Lebeidowska et al. (2009) for the hamstrings and rectus femoris (REF) in persons post-stroke and with CP [8]. Unfortunately, in these studies the distinction between the patterns was only described qualitatively. On the other hand, based on the idea that spasticity is related to a deregulation of stretch reflex thresholds (SRTs), Levin and Feldman (1994) measured dynamic SRTs (DSRTs) and used them to identify the tonic SRT (TSRT) in persons with elbow flexor spasticity [19]. DSRTs were defined as the joint angles at which electromyography (EMG), evoked by stretch at defined velocities, increased. By plotting the DSRTs on velocity-angle phase diagrams, a regression line was fitted to the data. Extrapolating this regression line to zero velocity allowed them to determine the TSRT, which represented the joint position beyond which motor unit recruitment would begin [19]. In comparison to healthy muscles, highly velocity-dependent DSRTs and a reduced TSRT were found in the elbow flexors of persons post-stroke [19], and in a later study, in children with CP [20]. When the TSRT occurred within the biomechanical joint range, voluntary relaxation and activation was limited and interfered with movement [21]. Apart from proving to be a reliable and valid way to assess spasticity, SRTs also present a way to understand the influence of velocity and length on individual muscles. In the triceps surae of subjects with spinal cord injury, Van der Salm et al. found that it was the position, rather than the velocity that determined the onset of pathological muscle activation [22]. Levin and Feldman (1994) reported that the amount of muscle activation would be proportional to the amount and rate of muscle lengthening [19]. This was confirmed by a study of Malhotra et al. (2008) who showed that muscles that were visually classified into activation patterns, also had significantly different EMG gain values with an increasing joint angle [16].

In daily clinical practice, commonly used spasticity assessment scales such as the Modified Ashworth Scale (MAS) [23], do not provide information on the underlying pathological muscle activation pattern during stretch [24]. Instead, in the aforementioned studies, muscle activation patterns have mostly been described using instrumented techniques that record biomechanical and electrophysiological signals during the stretch. By measuring kinematics while simultaneously registering muscle response using EMG, the instrumented methods are able to identify velocity and position thresholds and gain. Quantitative methods to assess activation patterns have received less attention in the lower-limb muscles of children with spastic CP. Recently, a manually-controlled instrumented spasticity assessment has been verified as psychometrically sound to quantitatively assess spasticity in the medial hamstrings (MEHs) and gastrocnemius (GAS) of children with CP by quantifying the increase in pathological muscle activation and joint torque with increasing stretch velocities [11]. By integrating biomechanical and electrophysiological data, this instrumented assessment also has the potential to record muscle activation patterns. Identifying muscle and subject-specific activation patterns in children with CP will lend insight into the pathophysiology of spasticity, and may eventually help to explain the observed treatment response variability.

Therefore, the aims of this study were to (1) describe the occurrence of muscle activation patterns in children with CP; (2) develop a visual classification method to identify activation patterns; (3) apply quantitative parameters that validate the use of this visual classification; and (4) check the reliability of the developed parameters. These aims are realised using a previously validated instrumented spasticity assessment [11] with quantitative parameters [20]. In addition, we aimed to expand the protocol of the instrumented spasticity assessment to four lower limb muscles (MEHs, GAS, REF and adductors -ADDs) as we hypothesised that spasticity patterns would be both muscle- and subject-specific.

## Materials and Methods

### Ethics Statement

Ethical approval was granted by the University Hospitals' Ethics Committee (B32220072814). Parents and subjects were informed of the procedure and provided written informed consent in accordance with the Declaration of Helsinki.

### Participants

Fifty-four children with spastic CP between the ages of 5 and 18 years participated in this study. Exclusion criteria were the presence of ataxia or dystonia, severe muscle weakness (<2+ on the Manual Muscle Test [25]), poor selectivity [26], bone deformities or contractures compromising the performance of pure single-plane muscle stretch, cognitive problems that could impede the measurements, previous lower limb orthopaedic surgery, intrathecal baclofen pump, selective dorsal rhizotomy, or BTX injections in the past 6 months.

### Measurement protocol

**Figure 1:**
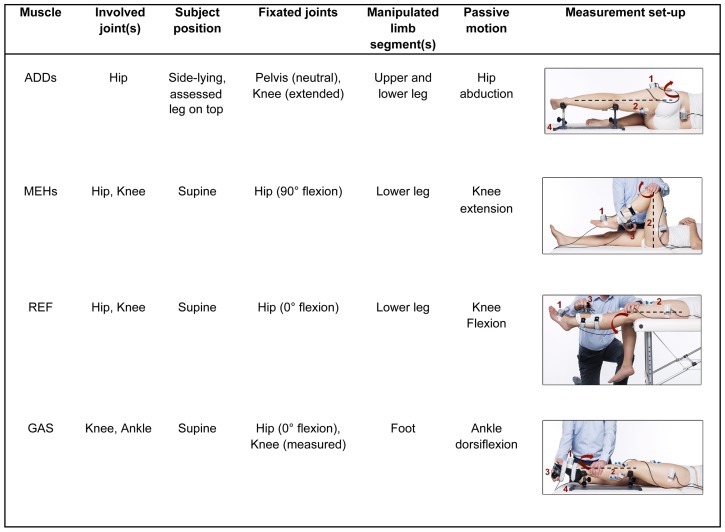
Measurement procedure for four lower limb muscles. ADDs, adductors; MEHs, medial hamstrings; REF, rectus femoris; GAS, gastrocnemius. The arrow indicates the direction of joint movement during stretch. Instrumentation: (1) two inertial measurement units (joint angle measurement); (2) surface electromyography (muscle activation measurement); and (3) a six DoF force-sensor attached to a shank or foot orthotic (torque measurement); (4) support frame.

All evaluations with the instrumented spasticity assessment were carried out by the same trained assessor. An overview of the measurement protocol per muscle can be found in Figure 1. Measurements of the MEHs and GAS have been previously described [11]. Stretches of the passive ADDs, MEHs, REF, and GAS, were performed by an examiner who moved one joint at a time (hip, knee, or ankle, respectively) while keeping non-moving joints fixated. For stretching the ADDs, hip abduction was performed with the subject in side-lying with the assessed leg on top, the knee extended, and the pelvis vertically aligned with the table ensuring no pelvic rotation. All other motions were performed in the sagittal plane with the patient in supine position. To stretch the MEHs and REF, knee flexion and extension were performed by manipulating a custom-made shank orthotic, strapped either to the posterior or anterior aspect of the lower leg, respectively. To stretch the GAS, ankle dorsiflexion was performed by manipulating a custom-made foot orthotic (see Figure 1). For each muscle, four stretch repetitions, at three velocities, over the full joint range of motion (ROM) were carried out. The hip, knee, or ankle was first moved at low velocity during 5 seconds, followed by intermediate, medium velocity over 1 second, and finally at high velocity, performed as fast as possible. The interval between each repetition was 7 seconds, to account for the effects of decreased post-activation depression.

The movement of the distal limb segment with respect to the proximal limb segment was tracked using two inertial measurement units (IMUs: Analog Devices, ADIS16354) that recorded angular velocity and acceleration. To compute the anatomical joint angles from IMU measurements, calibration trials with predefined motions were performed prior to the stretch trials. For the ADDs, a static calibration was carried out in side lying. The ankle and knee were supported by a frame, with the knee in extension, the hip joint positioned to zero degrees abduction, and the pelvis vertically aligned with the table ensuring no pelvic rotation. The calibration trials of the MEHs and GAS have been previously described [11]. For the REF and MEHs, the same calibration trial was used.

Throughout the measurement procedure, surface EMG from the four muscles and, in the case of the GAS, MEHs and REF, also their antagonists (tibialis anterior, REF, and MEHs, respectively), was collected using a telemetric Zerowire system (Cometa, Milan, IT) at a sample rate of 2000 Hz. Surface EMG electrodes were placed according to a standardized procedure and palpation [27]. Antagonist activation was used to detect other tone problems (e.g. dystonia) or active assistance of the child during stretches. Prior to stretching, three repetitions of isometric Maximum Voluntary Contractions (MVCs) were carried out per muscle with the child in supine. EMG data from these contractions were used as an individual reference to evaluate surface EMG signals measured during the passive stretch trials [11].

In addition to surface EMG and kinematics, joint torque was measured for the movements of the ankle and knee using a six degrees-of-freedom force/torque sensor load-cell attached to the orthotics (see Figure 1). Measurements of EMG, motion, and torque were synchronously captured in order to facilitate an integrated analysis. However, torque data were not analysed for the current study. More information on internal joint torque calculation can be found in [11].

A complete measurement of all four muscles on one side took half an hour. In children with unilateral CP, only the affected side was tested. In bilaterally involved children, if time permitted, both legs were assessed. If not, the most affected side was assessed (defined as the side with the highest averaged MAS score of the four muscles, or in case of symmetrical averaged MAS scores, the side with the most severe averaged Modified Tardieu angle [28]). For a group of ten children the full procedure was repeated (including replacement of all the sensors) after a rest interval of two hours (during which they received no treatment). These repeated measurements were used to evaluate the assessment's intra-rater reliability. In addition to instrumented spasticity assessments, another independent assessor performed a full clinical lower-limb assessment, including determination of spasticity by the MAS [23] and the Modified Tardieu angle [28].

### Data analysis

The root mean square (RMS) envelope of the surface EMG was computed using a low-pass 30-Hz 6^th^ order zero-phase Butterworth filter on the squared raw EMG signal. ROM and maximum angular velocity were obtained after applying a Kalman smoother [29] on the IMU-data. All stretch velocity profiles were bell-shaped. By visualizing the data, stretch repetitions were excluded when performed out of plane (see Supplement 1 in [11]), at inconsistent velocities between different repetitions within a velocity trial (difference >20°/s), in case of poor quality surface EMG (low signal-to-noise ratio or obvious artefacts), and in case of antagonist activation. Data visualization and analyses were carried out using custom software implemented in MATLAB (version 7.10.0 R2010a, MathWorks).

### Outcome parameters

**Figure 2:**
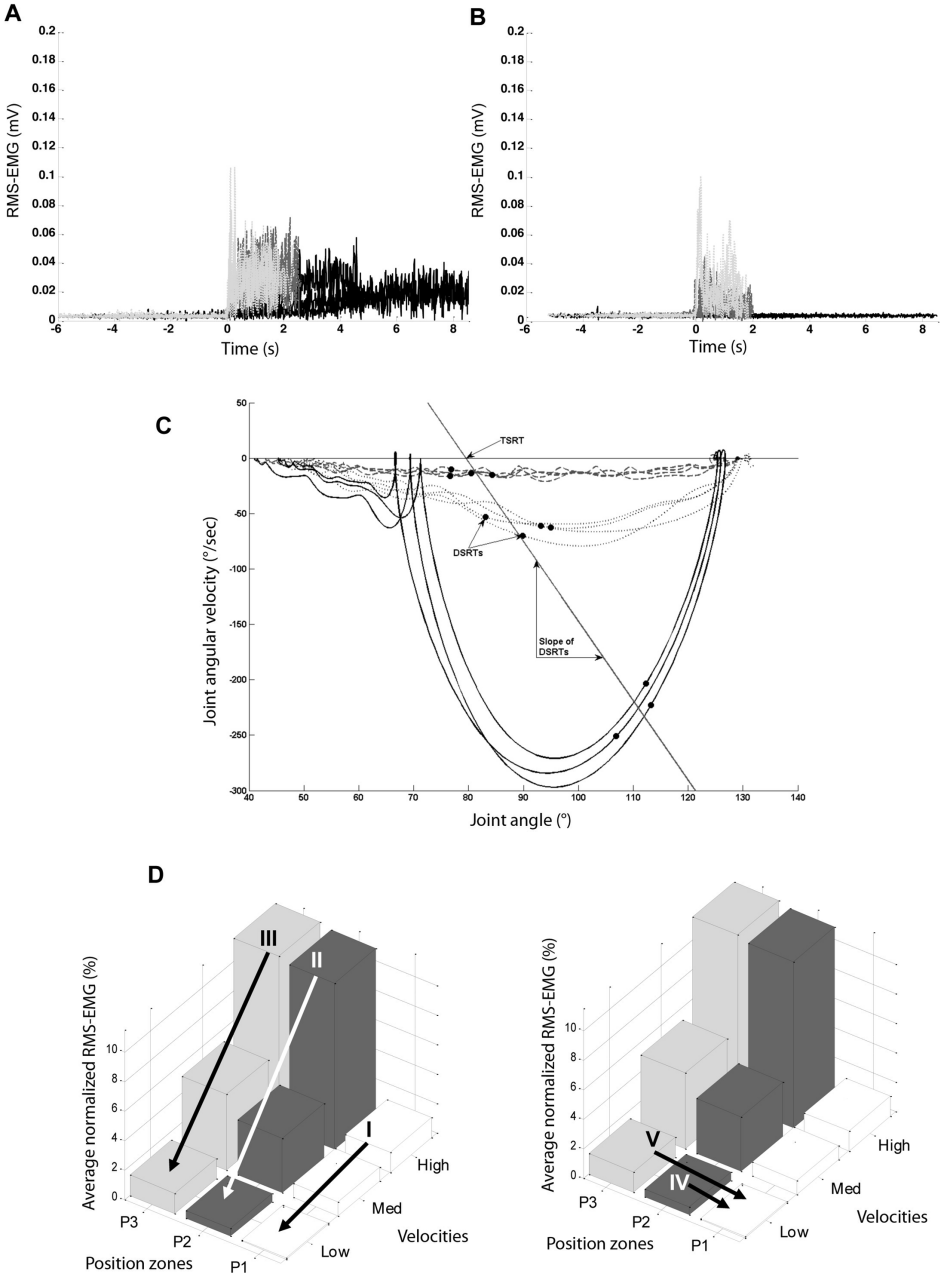
Graphs used during the visual categorization into patterns and for parameter development. Root mean square electromyography plotted versus time for medial hamstring during low (black), medium (grey, dashed) and, high (grey, dotted) velocity stretches. Zero seconds was expressed as the time that maximum velocity occurred. In A. a mixed low velocity-dependent, and in B. a high velocity-dependent activation pattern, is shown. C. *Dynamic stretch reflex thresholds* (DRSTs – dots) of the medial hamstrings in an angle-velocity phase graph at three stretch velocities: high (continuous line), medium (dotted line) and low (dashed line). The slope of a regression line through the DRSTs represents the sensitivity of reflexes to velocity [37]. The intersection of the regression line with the velocity-axis is defined as the *Tonic stretch reflex threshold (TSRT)* [37]. D. Average normalized RMS-EMG across three position zones (P1, P2, P3) and across three velocities (low, medium, high). I: Change in average normalized RMS-EMG in P1 (position zone 1: 10–36.6% of the ROM) between high and low velocity; II: Change in average normalized RMS-EMG in P2 (position zone 2: 36.6–63.3% of the ROM) between high and low velocity; III: Change in average normalized RMS-EMG in P3 (position zone 3: 63.3–90% of the ROM) between high and low velocity; IV: Change in average normalized RMS-EMG at low velocity between P1 and P2; V: Change in average normalized RMS-EMG at low velocity between P1 and P3.

Per velocity trial, the average maximum angular velocity was calculated per muscle. EMG onset was defined according to the method of Staude and Wolf [30]. This automatic onset detection method applies an approximated generalized likelihood principle by detecting statistically optimal changes throughout the signal [30], and has been shown to perform significantly better compared to threshold based algorithms [31]. In those cases when no onset was automatically detected due to the activation interval being too short, the onset could be visually determined on an RMS-EMG time graph (Figure 2A and B) viewed in a graphical user interphase of the same custom software. DSRTs, defined as the angles at EMG onset during the different stretch repetitions, were plotted on a joint angle-angular velocity phase graph as in (Figure 2C) [20]. When EMG onset occurred at all three stretch velocity conditions (allowing for a minimum of three data points) the slope of a linear regression through the DSRTs was calculated. This value represented the sensitivity of the reflexes to stretch [20]. The intersection of this regression line with the velocity-axis represented the estimated joint angle at which the muscle would be activated while the limb was at rest, previously defined as the TSRT [20], [32]. The TSRT was expressed as a percentage of the full ROM. This indicated where in the available ROM the TSRT would occur, and allowed for comparison between muscles and between subjects.

The effect of increasing velocity and joint angle on the gain in EMG was investigated by dividing each movement into three equal zones between 10–90% of the ROM. The zones were defined as the time windows corresponding to: 10–36.6% ROM (P1), 36.6–63.3% ROM (P2), and 63.3–90% ROM (P3). The time windows corresponding to the extremes of the ROM (<10% and >90%) were excluded as they appeared to be influenced by the performance of the therapist and the comfort of the patient. Average RMS-EMG per position zone was defined as the area underneath the RMS-EMG curve, divided by the duration of the corresponding position zone. These values were normalized by expressing them as a percentage of the peak RMS-EMG value of the three MVCs. One normalized RMS-EMG value per position zone at each velocity was calculated by averaging all stretch repetitions per velocity trial. These values were then plotted on a 3D bar graph (Figure 2D). The following parameters were created:

1. Within each position zone, the change in average normalized RMS-EMG between high and low velocity stretches (*EMG P1 high-low, EMG P2 high-low, and EMG P3 high-low*).
2. At low velocity, the change in average normalized RMS-EMG between P2 and P1 and between P3 and P1 (*EMG low P2-P1*, and *EMG low P3-P1*, respectively).

### Visual pattern categorization

**Figure 3:**
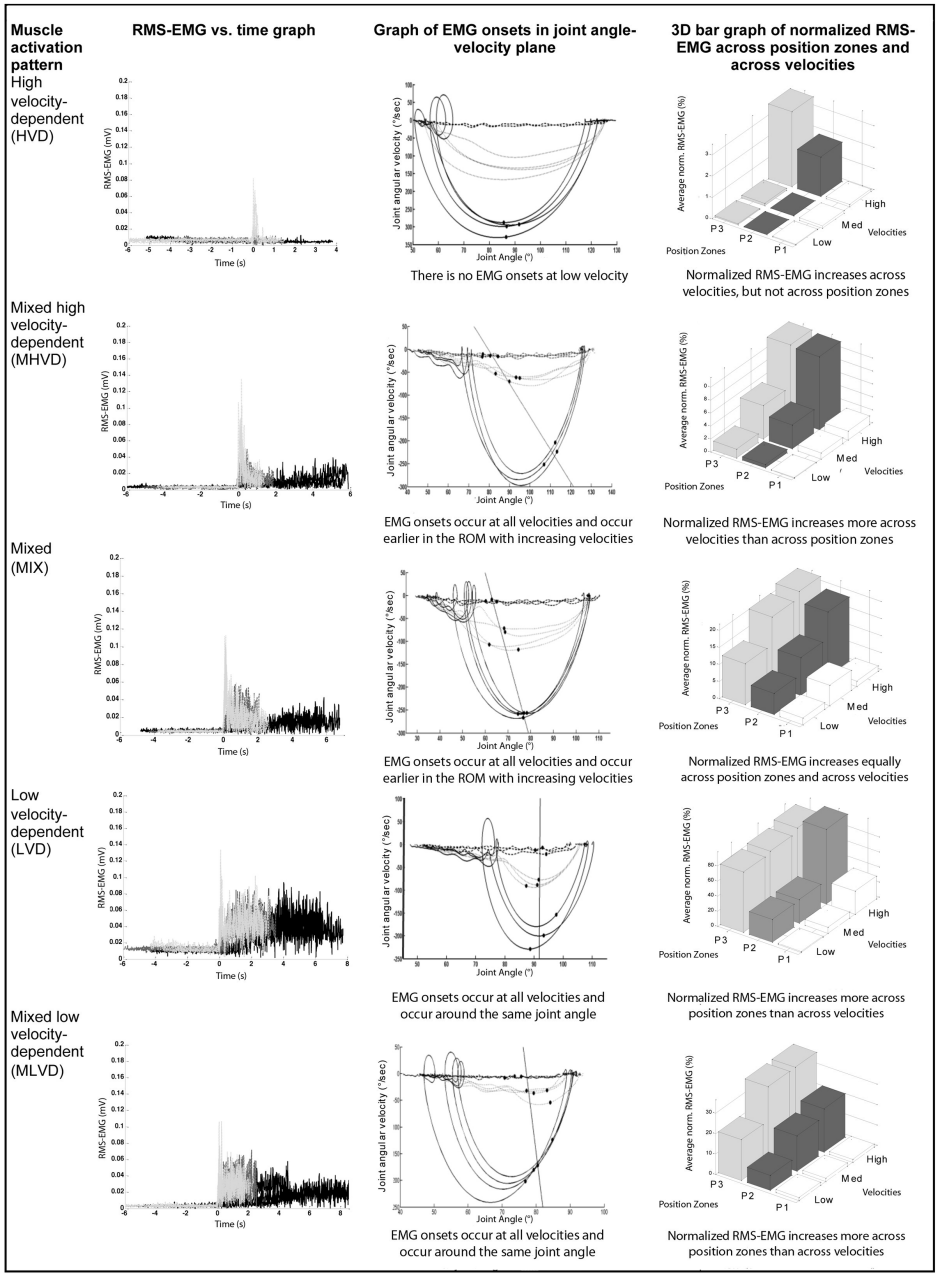
Examples of different activation patterns in the medial hamstrings. The graphs in the first, second and third column are further explained by Figures 2A, B, and C. EMG, electromyography; ROM, joint range of motion; RMS, root mean square.

Two researchers independently allocated each muscle to one of five possible activation patterns. When a disagreement occurred between the two researchers, a third was involved and the majority decision defined the final pattern for each muscle. The following criteria were used to classify muscles. Examples of graphs from each type of pattern can be found in Figure 3.

1. A muscle was categorized as having a high velocity-dependent (HVD) activation pattern when EMG onset was not automatically, or visually detected in the stretches performed during the low velocity trial, but was detected during the stretches performed at the high velocity trial. Additionally, average normalized RMS-EMG increased with higher stretch velocity.
2. A muscle was categorized as having a mixed high velocity-dependent (MHVD) activation pattern when EMG onset was automatically, or visually detected in all stretches performed during low, medium, and high velocity trials. EMG onset was detected earlier in the ROM the faster the velocity of the stretch. Average normalized RMS-EMG increased more with higher stretch velocity than with increasing ROM.
3. A muscle was categorized as having a mixed (MIX) activation pattern when EMG onset was automatically, or visually detected in all stretches performed during low, medium, and high velocity trials. EMG onset was detected earlier in the ROM the faster the velocity of stretch, but average normalized RMS-EMG increased as much with higher stretch velocity as with increasing ROM.
4. A muscle was categorized as having a low velocity-dependent (LVD) activation pattern when EMG onset was automatically, or visually detected around the same joint angle in all stretches performed during low, medium, and high velocity trials. Average normalized RMS-EMG increased with increasing ROM and was unaffected by higher velocity.
5. A muscle was categorized as having a mixed low velocity-dependent (MLVD) activation pattern when EMG onset was automatically or visually detected in all stretches performed during low, medium, and high velocity trials. EMG onset was either detected earlier in the ROM with faster stretch velocity, or onsets were centred around one joint angle. Average normalized RMS-EMG increased more with increasing ROM than with higher stretch velocity.

### Statistical analysis

Percentage exact agreement between researchers to visually classify the activation patterns was calculated. Freeman Holton tests were used to assess whether the final allocation to different activation patterns differed significantly between muscles. Intra-rater reliability of the developed parameters was assessed using intraclass correlation coefficients (ICC1,1) [33] with 95% confidence intervals and the standard error of measurement (SEM). The SEM was calculated from the square root of the mean square error from one-way ANOVA [34]. ICC-values 0.80 indicated high; 0.60 moderately high; and 0.40 moderate reliability [35]. Face validity of the visual classification was tested by comparing the developed parameters between muscles categorized into activation patterns using either t-tests, or in case of more than two categories, ANOVA and post-hoc Tukey tests. In addition, age, gender, and anatomic distribution of the motor impairment (unilateral vs. bilateral involvement) of the children whose muscles were classified into different activation patterns were compared per muscle using similar statistical tests (continuous parameters), or Chi Square tests (categorical parameters). Significance was set at *p*<0.05. All statistical analyses were carried out in SPSS (IBM Statistics 20).

## Results

Fifty-four children, 36 males and 19 females, participated in the study (Table 1). Due to time-restrictions, not all subjects underwent instrumented spasticity assessments in all four muscles. In bilaterally involved children, both sides were tested on 7 occasions for the MEHs, 3 times for the ADDs and the GAS, and once for the REF. Four ADDs, 7 GAS, 3 MEHs and 2 REF were not classifiable and were therefore excluded for further data analysis. These muscles could not be classified because of: absence of any EMG activity at any velocity, poor EMG quality, or an unrecognizable and inconsistent pattern which was judged as being affected by the performance of the measurement. In total, 28 ADDs, 44 GAS, 55 MEHs and 34 REF muscles were analysed. EMG onset was visually determined in 64 of the total 318 ADD stretch repetitions, in 40 of the 492 GAS stretch repetitions, in 38 of the 658 MEH stretch repetitions, and in 46 of the 392 REF stretch repetitions.

**Table 1:** Patient's characteristics.

Characteristics	Subjects (n = 54)					Subjects reliability study (n = 10)				
Age (mean ± SD)	10.9yrs ±3.9 yrs					11.9yrs ±3.8yrs				
Gender (n)	36 Males; 18 Females					7 Males; 3 Females				
Level of involvement (n)	22 Unilateral (11 RH; 11 LH) 32 Bilateral (28 Di; 2 Tri; 2 Quad)					4 Unilateral (2 LH; 2 RH) 6 Bilateral (5 Di; 1 Quad)				
GMFCS level I–IV (n)	I: 32; II: 15; III: 6; IV: 1					I: 5; II: 4; III: 0; IV: 1				
MAS score 0–5	0	1	1+	2	3	0	1	1+	2	3
MAS ADDs (n)	8	7	4	7	2	1	5	2	2	0
MAS MEHs (n)	2	8	23	16	6	0	0	2	7	1
MAS GAS (n)	0	4	18	18	4	0	0	4	4	2
MAS REF (n)	13	11	5	4	1	3	2	2	2	1

**Abbreviations:** RH, right hemiplegia; LH, left hemiplegia; Di, diplegia; Tri, triplegia; Quad, quadriplegia; GMFCS, Gross Motor Function Classification Score; MAS, Modified Ashworth Scale; ADDs, adductors; GAS, gastrocnemius; MEHs, medial hamstrings; REF, rectus femoris.

Percentage exact agreement between assessors to categorise muscles into activation patterns ranged from 83% to 97%. An overview of the final pattern categorization can be found in Table 2. ADDs, GAS and REF were categorized as MHVD or HVD. One MEHs muscle was classified as MIX and one as LVD. The rest of the MEHs were classified as HVD, MHVD or, MLVD. There were significantly more GAS and REF muscles categorized as HVD than MEHs (*p*<0.001). Among MHVD patterns, there were significantly more ADDs and MEHs muscles than GAS and REF (*p*<0.001). To allow for group comparisons, the muscle with an LVD pattern was added to the MLVD group and the muscle with a MIX pattern was added to the MHVD group.

**Table 2:** Allocation of muscles to activation patterns based on visual categorization.

Activation pattern	MIX	MLVD	MHVD	HVD	LVD	PEA
Muscle						
ADDs	0	0	20	8	0	85.71%
GAS	0	0	13	31	0	72.73%
MEHs	1	7	34	12	1	83.64%
REF	0	0	7	27	0	97%
*p-value	NR	NR	<0.001	<0.001	NR	NR

**Note:** Percentage Exact Agreement (PEA) of two independent assessors. The final allocation was based on majority decision with involvement of a third independent assessor.

**Abbreviations:** ADDs, adductors; GAS, gastrocnemius; MEHs, medial hamstrings; REF, rectus femoris; MIX, mixed; MHVD, mixed, high velocity-dependent; MLVD, mixed, low velocity-dependent; HVD, high velocity-dependent, LVD, low velocity-dependent; PEA, percentage exact agreement; NR, not relevant. *Freeman Holton tests for significantly different allocation of muscles to HVD and MHVD patterns *p*<0.05.

The reliability results of all outcome parameters can be found in Table 3. One ADDs trial from the reliability study was excluded due to bad quality EMG data. The reliability of the slope of the DSRTs and of the value of the TSRT could only be calculated in those muscles with an EMG onset at low velocity (in 8 of the 10 ADDs and MEHs). Relative reliability values were moderate to high (ICC 0.45–0.97). The SEM values tended to be lower for parameters of the MEHs and GAS than for the ADDs and REF.

**Table 3:** Averages and standard deviations (SD) of parameters of the adductors (ADDs) and gastrocnemius (GAS) in both sessions (test, retest) and intra-class correlation coefficients (ICC) and standard error of measure (SEM) for intra-rater reliability.

	ADDs (n = 9)				GAS (n = 10)			
	Test	Retest	ICC	SEM	Test	Retest	ICC	SEM
V_MAX_ low (°/s)	11.52 (2.76)	10.87 (3.68)	0.54	2.66	16.46 (5.99)	15.09 (7.73)	0.94	2.24
V_MAX_ med (°/s)	49.08 (9.31)	40.27 (7.26)	−0.07	7.53	66.52 (19.01)	68.22 (16.34)	0.74	12.02
V_MAX_ high (°/s)	102.52 (19.82)	88.74 (16.71)	0.62	11.28	163.50 (30.97)	158.32 (18.85)	0.90	10.65
ROM (°)	19.82 (37.64)	16.71 (33.37)	0.48	7.17	51.53 (8.77)	50.27 (6.68)	0.86	3.93
EMG P1 high-low (%)	7.13 (6.95)	6.30 (5.82)	0.69	4.56	0.79 (2.19)	0.56 (1.83)	0.51	1.69
EMG P2 high-low (%)	12.30 (9.30)	10.68 (7.53)	0.82	4.75	13.54 (9.60)	15.38 (15.53)	0.88	6.13
EMG P3 high-low (%)	10.09 (5.39)	9.93 (6.60)	0.80	3.63	7.09 (5.76)	5.24 (6.97)	0.61	4.83
EMG low P2-P1 (%)	0.93 (1.00)	1.26 (1.84)	0.86	0.75	0.21 (0.36)	0.39 (0.57)	0.81	0.22
EMG low P3-P1 (%)	3.55 (3.28)	3.71 (4.10)	0.75	2.48	1.59 (2.36)	1.57 (2.11)	0.69	1.60
Slope of the DSRTs (°/s) (n = 8)	−0.28 (0.13)	−0.35 (0.20)	0.75	0.10	NR	NR	NR	NR
TSRT (°) (n = 8)	18.27 (11.72)	14.71 (9.49)	0.91	3.74	NR	NR	NR	NR

	MEHs (n = 10)				REF (n = 10)			
V_MAX_ low (°/s)	17.46 (7.65)	17.61 (6.36)	0.86	3.59	19.81 (6.18)	18.48 (8.18)	0.96	2.00
V_MAX_ med (°/s)	98.84 (30.09)	102.26 (25.06)	0.71	19.21	107.65 (33.08)	98.61 (30.08)	0.90	13.52
V_MAX_ high (°/s)	265.05 (39.18)	263.14 (41.17)	0.89	18.34	249.01 (34.47)	246.70 (33.98)	0.68	25.17
ROM (°)	69.84 (13.15)	72.82 (14.34)	0.96	3.09	86.83 (10.23)	86.74 (15.76)	0.91	5.80
EMG P1 high-low (%)	4.49 (5.04)	2.09 (3.35)	0.45	3.46	10.60 (17.60)	11.66 (20.23)	0.57	15.44
EMG P2 high-low (%)	17.16 (9.72)	18.65 (9.65)	0.76	6.21	35.21 (28.82)	46.90 (46.63)	0.78	22.08
EMG P3 high-low (%)	16.97 (12.88)	14.12 (6.01)	0.76	6.21	24.52 (18.82)	27.70 (25.73)	0.86	11.71
EMG low P2-P1 (%)	1.87 (2.33)	1.81 (2.06)	0.84	1.20	0.16 (0.39)	0.27 (0.51)	0.70	0.32
EMG low P3-P1 (%)	4.42 (4.81)	5.11 (5.63)	0.89	2.43	0.62 (0.95)	0.96 (1.65)	0.44	1.18
Slope of the DSRTs (°/s) (n = 8)	−0.07 (0.11)	−0.05 (0.06)	0.89	0.04	NR	NR	NR	NR
TSRT (°) (n = 8)	83.13 (15.49)	83.11 (14.73)	0.97	4.06	NR	NR	NR	NR

**Abbreviations:** V_MAX_, maximum angular velocity; low, low velocity stretches; high, high velocity stretches; ROM, range of motion; EMG, electromyography; P1, position zone 1; P2, position zone 2; P3, position zone 3; DSRT, dynamic stretch reflex threshold; TSRT, tonic stretch reflex threshold.

Most of the developed outcome parameters were significantly different between activation patterns highlighting good face validity (Table 4 and 5). Two parameters (EMG low P2-P1, and EMG low P3-P1) were able to distinguish between all patterns in all muscles (*p*<0.01). The slope of the DSRTs and TSRT were not calculated for HVD patterns as they required an EMG onset at low velocity. In the MEHs, the median slope of the DSRTs in MHVD patterns was significantly steeper (*p* = 0.002), and the TSRT occurred significantly later in the ROM (*p* = 0.001) than in MLVD patterns. Children with GAS muscles categorized as MHVD were younger than those with a HVD pattern (*p* = 0.002). Children with MEHs muscles classified as MHVD or MLVD were more likely to be bilaterally involved, while the children with MEHs muscles classified as HVD often had a unilateral involvement (*p* = 0.009) (Table 5).

**Table 4:** Means and (SD) of outcome parameters and patient characteristics for the adductors (ADDs) and gastrocnemius (GAS) – comparison within each muscle between activation patterns.

	ADDs			GAS		
Parameters	MHVD (n = 20)	HVD (n = 8)	*p*	MHVD (n = 13)	HVD (n = 31)	*p*
V_MAX_ low (°/sec)	12.70 (3.58)	13.92 (3.63)	0.45	18.60 (5.48)	18.35 (4.39)	0.87
V_MAX_ high (°/sec)	108.30 (28.51)	130.61 (44.90)	0.13	164.27 (23.97)	168.30 (32.33)	0.69
ROM (°)	41.41 (11.68)	47.43 (15.81)	0.28	54.21 (11.11)	53.86 (9.87)	0.91
EMG P1 high-low (%)	7.38 (7.61)	11.58 (18.15)	0.39	1.04 (1.60)	0.21 (1.52)	0.11
EMG P2 high-low (%)	12.12 (9.41)	3.67 (4.40)	0.02**	13.34 (6.04)	7.45 (7.56)	0.02**
EMG P3 high-low (%)	11.69 (9.46)	4.64 (3.82)	0.53	8.00 (5.21)	3.62 (4.37)	0.01**
EMG low P2-P1 (%)	1.02 (1.18)	<0.01 (0.47)	0.03**	0.53 (0.71)	0.10 (0.32)	0.01**
EMG low P3-P1 (%)	3.84 (4.13)	0.31 (0.86)	0.03**	3.00 (2.26)	0.45 (0.69)	<0.01**
Slope of DSRTs (°/s)	0.29 (0.19)	NR	NR	0.06 (0.03)	NR	NR
TSRT % ROM (%)	76.42 (21.10)	NR	NR	58.67 (11.20)	NR	NR
Age (years)	11.59 (3.83)	10.92 (4.02)	0.68	8.57 (2.83)	12.19 (3.45)	<0.01**
Gender: male/female (n)	13/7	4/4	0.46	8/5	23/8	0.40
Unilateral/bilateral involvement (n)	7/13	2/6	0.61	3/10	14/17	0.17

**Abbreviations:** ADDs, adductors; GAS, gastrocnemius; MEHs, medial hamstrings; REF, rectus femoris; MHVD, mixed, high velocity-dependent; HVD, high velocity-dependent; MLVD, mixed, low velocity-dependent; V_MAX_, maximum angular velocity; low, low velocity stretches; high, high velocity stretches; ROM, range of motion; EMG, electromyography; P1, position zone 1; P2, position zone 2; P3, position zone 3; DSRTs, dynamic stretch reflex thresholds; TSRT, tonic stretch reflex threshold.

^**^Significant difference: p<0.05 (t-test/Chi square).

**Table 5:** Means and (SD) of outcome parameters and patient characteristics for the medial hamstrings (MEHs) and rectus femoris (REF) – comparison within each muscle between activation patterns.

	MEHs				REF			
Parameters	MLVD (n = 8)	MHVD (n = 35)	HVD (n = 12)	*p*		MHVD (n = 7)	HVD (n = 27)	*p*
V_MAX_ low (°/sec)	21.85 (10.37)	21.95 (5.90)	20.66 (3.70)	0.83		26.26 (8.40)	22.18 (5.66)	0.13
V_MAX_ high (°/sec)	239.26 (5.40)	283.26 (43.68)	310.08 (27.89)	<0.01*		230.67 (45.46)	252.90 (29.37)	0.12
ROM (°)	67.63 (19.73)	77.7 (9.52)	81.32 (7.77)	0.03		85.64 (16.99)	89.02 (9.39)	0.48
EMG P1 high-low (%)	4.46 (10.59)	4.32 (3.67)	0.80 (1.29)	0.10		8.75 (9.54)	7.81 (11.38)	0.84
EMG P2 high-low (%)	29.19 (22.72)	22.82 (13.51)	7.93 (6.23)	<0.01* b ^,^ c		55.64 (57.21)	30.77 (39.95)	0.19
EMG P3 high-low (%)	10.44 (4.41)	16.65 (12.03)	8.81 (6.64)	0.05		36.34 (36.80)	20.64 (23.85)	0.18
EMG low P2-P1 (%)	8.26 (8.82)	1.38 (1.74)	0.10 (0.27)	<0.01* a ^,^ c		10.69 (16.15)	−0.03 (0.23)	<0.01**
EMG low P3-P1 (%)	23.47 (24.79)	4.24 (4.24)	0.25 (0.50)	<0.01* a ^,^ b ^,^ c		11.17 (11.43)	0.09 (0.44)	<0.01**
Slope of DSRTs (°/s)	0.02 (0.05)	0.10 (0.08)	NR	0.01**		0.10 (0.08)	NR	NR
TSRT % ROM (%)	30.48 (9.23)	58.22 (10.10)	NR	<0.01**		47.07 (17.04)	NR	NR
Age (years)	11.00 (4.13)	10.38 (3.33)	11.25 (3.53)	0.72		10.03 (3.77)	11.46 (3.72)	0.37
Gender: male/female (n)	5/3	24/11	7/5	0.80		5/2	17/7	0.68
Unilateral/bilateral involvement (n)	0/8	12/23	8/4	<0.01* b ^,^ c		2/5	11/16	0.56

**Abbreviations:** ADDs, adductors; GAS, gastrocnemius; MEHs, medial hamstrings; REF, rectus femoris; MHVD, mixed, high velocity-dependent; HVD, high velocity-dependent; MLVD, mixed, low velocity-dependent; V_MAX_, maximum angular velocity; low, low velocity stretches; high, high velocity stretches; ROM, range of motion; EMG, electromyography; P1, position zone 1; P2, position zone 2; P3, position zone 3; DSRTs, dynamic stretch reflex thresholds; TSRT, tonic stretch reflex threshold.

*Significant difference: p<0.05 (ANOVA/Freeman Holton).

^**^Significant difference: p<0.05 (t-test/Chi square).

a Significant difference between MHVD and MLVD (Post-hoc Tukey test/Chi Square).

b Significant difference between MHVD and HVD (Post-hoc Tukey test/Chi Square).

c Significant difference between MLVD and HVD (Post-hoc Tukey test/Chi Square).

## Discussion

This is the first study to report and quantitatively assess different muscle activation patterns during passive stretching of lower-limb muscles in a large number of children with spastic CP. In addition, we are the first to report on the reliability of quantitative parameters that can distinguish between patterns in the lower-limb muscles of children with CP. The velocity profiles and EMG onsets were repeatable both in the individual muscle (see Figures 2 and 3) and on a group analysis (Table 3). The relative intra-rater reliability of the TSRT in the MEHs and ADDs was higher than that reported by Calota et al. who used a similar hand-held device to calculate the TSRT in elbow flexors of persons post-stroke. It was also higher than that reported by Jobin and Levin (2000) who applied a torque motor to stretch the muscles of children with CP [20]. In the latter studies, EMG onset was automatically defined as the point at which the EMG signal increased 2SDs above the mean baseline EMG. This automatic onset detection method is inaccurate in situations of any baseline noise or gradual onset rise time [30]. Although more robust than the threshold method, the automatic detection method applied in the current study failed to detect any activation in 10% of all stretch repetitions. In these cases, onset was visually determined which may have contributed to the higher reliability. While visual determination is considered to provide accurate event detection due to the signal being assessed by an expert, it is still subjective and time consuming.

In order to highlight true differences, it is important that the system's measurement error is smaller than the average differences between patterns. The information from this study proves promising for carrying out a sensitivity analysis to compare alterations in muscle activation patterns over time, or after treatment. However, in the current study, the limited number of subjects used to assess reliability, especially for the TSRT, and the visual determination of EMG onset in 10% of the stretch repetitions, necessitates caution when interpreting the results.

Assessing spasticity using instrumented measurements has been found superior to clinical spasticity assessments [5]. Quantitative interpretation of data by integration of muscle stretch characteristics with EMG provided a visual as well as quantified way to highlight low or high velocity-dependent muscle activation. We applied previously-developed parameters that captured the sensitivity of reflex thresholds, and EMG gain. Both components are important contributors to spasticity severity. Thresholds represent the initiators of motor neuron recruitment (hyper-excitability) while EMG gain represents the number of motor neurons recruited (hypersensitivity). However, developing parameters that quantify reflex thresholds and gain, presents some methodological challenges. Wu et al. have shown that spasticity with velocity-dependency may also be partly due to position change because the joint is moved further in the ROM at higher velocities [36]. Secondly, applying manual stretches results in inconsistencies in velocity. These two issues confound the direct comparison of absolute EMG threshold joint angles between subjects and between muscles. Calculation of the slope of the DSRTs and the TSRT (as a percentage of the ROM) helped to overcome these issues. The slope of the DSRTs was found to be steeper and the TSRT later in the ROM in MHVD than in MLVD patterns. Calota et al. found that manual stretches at variable velocities are preferred for calculation of the TSRT [37]. In their study, the TSRT was more difficult to locate in muscles with low spasticity where the DSRT values were either widely dispersed due to faulty EMG onset detection, or only a limited number of DSRT values could be identified. Similarly, in the current study, the TSRT could not be calculated in pure HVD patterns, which may also be considered to reflect low levels of spasticity.

EMG gain is known to be velocity-dependent [9], [11]. This was confirmed in the current study by the existence of some velocity-dependent increase in EMG gain in all the studied muscles. However, in muscles categorised as MHVD and MLVD, EMG gain also increased with increasing muscle length even when stretch velocity was low. Similar, longer duration, tonic activations have been reported by other authors during low velocity stretches of spastic muscles in adults [8], [9], [22], [38]. Two of the developed EMG gain parameters successfully distinguished between all patterns in all muscles. These were: the change between position zones 1 and 2, and between position zones 1 and 3 at low velocity. The higher these values, the lower the activation threshold. Furthermore, the SEM values for these two parameters for all muscles, and for the slope of the DSRTs of the MEHs, were sufficiently low to detect differences between activation patterns. Malhotra et al. (2008) identified pure LVD activation patterns in some spastic wrist flexors post-stroke, whereby there was no influence of increasing velocity on EMG gain [16]. Such a pattern was only found in one MEHs muscle in the current CP cohort, and confirms the finding that velocity-sensitivity is higher in children with CP than in persons post-stroke [20].

While it was not possible to explore the exact pathophysiological basis for the variations in the muscle activation patterns, possible contributing mechanisms may be considered. Pure HVD activation patterns may be related to the velocity sensitivity of Ia afferents and decreased central control (e.g. decreased presynaptic inhibition on Ia afferent pathways) [12]. LVD activation may be related to changes in the membrane properties, PIC, and the creation of plateau potentials in spinal neurons [13]. Some authors have also suggested that LVD activation reflects hypersensitivity of type II muscle spindle afferents [12], [16]. However, histological results regarding fibre type distribution and transformation due to spasticity are inconclusive [39]. More conclusive are the findings of altered muscle properties in spastic versus healthy muscles; such as increased muscle cell stiffness, and decreased quality of the extracellular matrix [40]. These changes result in stiffer muscles that are less compliant. Since the discharge rate of muscle spindles is dependent on absolute, as well as relative fibre length, and the velocity of fibre movement [18], [41], stiffer muscles may affect spindle hypersensitivity, possibly due to increased fusimotor activation [42]. This may help explain why in LVD and MLVD activation patterns, the gain in RMS-EMG was sensitive to increasing muscle length. On the other hand, Dietz and Sinkjaer (2007) suggested, that changes in the muscle properties might also influence the stretch reflex behaviour via non-spindle mechanoreceptors, such as pain-related group III/IV sensory muscle afferents [43].

The current study provides evidence of a large variability in the amount of activation and patterns among subjects. Similarly, Lebiedowska at al. (2009) also reported a larger heterogeneity of muscle activation patterns in response to stretch among subjects with CP compared to patients post-stroke [8]. In the current study, children who had an MHVD pattern in their GAS tended to be younger than those categorized as HVD. Additionally, children who had mixed patterns in their MEHs were more likely to be bilaterally involved. The link between certain patterns and patient or pathology characteristics should be further investigated in larger samples.

The classification of activation patterns was also found to be muscle-specific. The ADDs and the MEHs had a greater tendency towards MHVD; the GAS and REF were more HVD. MLVD patterns were only present in the MEHs. The amount of muscle stretch, and therefore the number and type of activated muscle spindles, will depend on fibre arrangement, length, orientation and, as previously described, muscle extensibility [42]. Therefore, our finding that different activation patterns occur in different muscles was not unexpected. Additionally, several studies have reported length dependent activation described by findings of a relationship between the starting muscle length and the appearance of SRTs during passive stretch [18], [21], [41]. This relationship may also be muscle specific. The REF and GAS were found to be less sensitive when stretched from initially longer lengths [44], whilst in the hamstrings, the opposite was reported [12]. In bi-articular muscles, the position of both joints is important when considering length dependency [21]. It is therefore possible that in the current study, due to the flexed hip at starting position, the MEHs were already being partly stretched from an elongated initial position, therefore increasing the likelihood of the SRT being reached faster. Since a similar starting position is applied during a clinical evaluation of the hamstrings (knee ROM, MAS, and Modified Tardieu angle), clinicians should be careful not to mistake MLVD activation with the evaluation of contracture.

The results of this study open many avenues for future clinical and research investigations. Given the large treatment response variability among children with CP to treatment with BTX [5], an investigation into whether the type of activation pattern present affects treatment outcome, is warranted. Secondly, identifying muscle-specific patterns may help in the development of more targeted treatment modalities. For example, a longer casting period may be recommended for MLVD muscles. Thus far, the muscle activation patterns described in literature do not seem to be related to the amount or shape of joint torque produced as the passive muscle is lengthened [16]. Nevertheless, a comprehensive assessment of spasticity should also include an evaluation of resistance to muscle stretch. Differentiation between the neural and non-neural contributions to increased joint torque during muscle stretch is essential to effectively distinguish spasticity from contracture. Therefore, assessments should be expanded to investigate how different activation patterns specifically contribute to the measured joint torque. Finally, as the ultimate goal of spasticity management is to improve function, the extent to which the existence of different activation patterns are related to abnormal voluntary movement and gait patterns should be further investigated [21].

To conclude, different muscle activation patterns were identified in four lower limb muscles of children with spastic CP. Activation patterns were found to be subject and muscle-specific. These differences can best be quantified by parameters that highlight the effect of increased muscle lengthening on the gain in EMG, during low velocity stretches. Such parameters were reliable, contained a low measurement error, and were sensitive to distinguish between different activation patterns in subjects and muscles. Information on the type, and quantification of the different activation patterns, may be useful in explaining response variability and directing spasticity treatment.
